# Impairment of Autophagic Flux After Hypobaric Hypoxia Potentiates Oxidative Stress and Cognitive Function Disturbances in Mice

**DOI:** 10.1007/s12264-023-01099-6

**Published:** 2023-08-22

**Authors:** Shuhui Dai, Yuan Feng, Chuanhao Lu, Hongchen Zhang, Wenke Ma, Wenyu Xie, Xiuquan Wu, Peng Luo, Lei Zhang, Fei Fei, Zhou Fei, Xia Li

**Affiliations:** 1 Department of Neurosurgery, Xijing Hospital, Fourth Military Medical University, Xi’an, 710000 China; 2 National Translational Science Center for Molecular Medicine and Department of Cell Biology, Fourth Military Medical University, Xi’an, 710000 China; 3 Department of Neurosurgery, The Central Hospital of Baoji, Baoji, 721000 China; 4 Department of Ophthalmology, Xijing Hospital, Fourth Military Medical University, Xi’an, 710000 China

**Keywords:** Autophagy, Proteomics, Oxidative stress, Hypobaric hypoxia, Brain injury

## Abstract

Acute hypobaric hypoxic brain damage is a potentially fatal high-altitude sickness. Autophagy plays a critical role in ischemic brain injury, but its role in hypobaric hypoxia (HH) remains unknown. Here we used an HH chamber to demonstrate that acute HH exposure impairs autophagic activity in both the early and late stages of the mouse brain, and is partially responsible for HH-induced oxidative stress, neuronal loss, and brain damage. The autophagic agonist rapamycin only promotes the initiation of autophagy. By proteome analysis, a screen showed that protein dynamin2 (DNM2) potentially regulates autophagic flux. Overexpression of DNM2 significantly increased the formation of autolysosomes, thus maintaining autophagic flux in combination with rapamycin. Furthermore, the enhancement of autophagic activity attenuated oxidative stress and neurological deficits after HH exposure. These results contribute to evidence supporting the conclusion that DNM2-mediated autophagic flux represents a new therapeutic target in HH-induced brain damage.

**Supplementary Information:**

The online version contains supplementary material available at 10.1007/s12264-023-01099-6.

## Introduction

Acute high-altitude (HA) exposure remains one of the main safety issues for tourism and those who enter high plateaus, making acute mountain sickness (AMS) a public health problem [1]. With increasing altitude, the partial pressure of inspired oxygen (O_2_) decreases, resulting in less O_2_ transport to the organs and tissues. The relative fall in tissue O_2_ levels induced by low barometric pressure leads to cellular hypoxia, which is a common cause of many altitude diseases [2]. The brain is highly sensitive to hypoxia manifested as brain edema, blood-brain barrier damage, and neuronal loss [3]. Acute hypobaric hypoxic brain damage is characterized by headache, cognitive deficits, and even high-altitude cerebral edema (HACE) which may become fatal [4]. As the primary energy source of neuronal activity is oxidative phosphorylation at the inner mitochondrial membrane in the form of ATP [5], hypoxia poses a challenge to O_2_ absorption, consumption, and delivery. When aerobic capacity is limited due to high altitude, organelles such as mitochondria can become a target of hypoxia which causes a sharp increase in the production of reactive O_2_ species (ROS), leading to oxidative stress and subsequent neuronal death [6].

Autophagy (referred to here as macroautophagy) is an intracellular lysosomal degradation pathway responsible for recycling nutrients to provide bioenergetic intermediates and maintain cellular homeostasis against external stress [7, 8]. Autophagosomes engulf cytoplasmic proteins and damaged organelles and deliver them to the lysosome. The defects in autophagy-lysosomal function are responsible for neuronal damage in multiple central nervous system diseases [9]. Over the past few decades, autophagy has been shown to be beneficial in preclinical research, including lifespan extension, improvement in neuronal function in aged animals, enhancement of tumor suppression, and defense against metabolic diseases [10–12]. A recent study has suggested that autophagy alleviates diquat-induced oxidative stress in IPEC-J2 cells and human periodontal ligament cells [13, 14]. However, the role of autophagy in acute hypobaric hypoxic brain damage and its regulatory mechanism is not understood.

In the present study, we simulated HA exposure in a decompression chamber and aimed to elucidate the role of autophagy and the molecular mechanisms underlying the regulation of autophagic activity in oxidative brain damage. The data-independent acquisition (DIA) proteomic was utilized to screen the potential key proteins related to HH-induced autophagy and it was found that DNM2 has an important role in maintaining autophagic flux. Our findings provided novel insights into the pathophysiological process of acute hypobaric hypoxic brain damage.

## Materials and Methods

### Animals and Experimental Design

A total of 155 C57BL/6J male mice (6–8 weeks old) and 24 mGFP-RFP-LC3 transgenic mice were used in the study. All procedures were performed according to guidelines published by the National Institutes of Health (No. 8023) and approved by the Institution Animal Care and Use Committee of the Fourth Military Medical University. Animals were housed in a temperature-controlled room (23 ± 2ºC) with a 12 h light/dark cycle with free access to food and water. The experimental design and group assignment were shown in Fig. S1 and described as follows:

In experiment 1, considering the possible fatal damage of HH exposure to mice, 59 C57BL/6J mice were randomly divided into four groups including the normoxia group (NG, *n* = 9) and HH groups (HH) of 4 (*n* = 15), 6 (*n* = 15) and 8 (*n* = 20) km altitude. The mice in the NG group were kept in the normobaric normoxic condition. Behavioral tests were performed in the normoxic room on days 1, 3, and 7 after HH exposure and sacrificed for further afterward.

In experiment 2, the mice exposed to 6 km altitude for 7 days were used for the oxidative stress and proteomics analysis.

In experiment 3, six mGFP-RFP-LC3 mice and 24 C57BL/6J mice were exposed to 6 km altitude for 7 days, and six mGFP-RFP-LC3 mice were assigned to the NG group. The 24 C57BL/6J mice were divided into two groups randomly and evenly including HH+blank dissolvent (HH+Veh) and HH+Rapamycin (HH+Rapa) groups.

In experiment 4, 12 mGFP-RFP-LC3 mice were divided into two groups (AAV2/9-hSyn-NC or AAV2/9-hSyn-DNM2 transfection) before HH exposure. 54 C57BL/6J mice were divided into two groups (AAV2/9-hSyn-NC or AAV2/9-hSyn-DNM2 transfection) and in each group, 27 mice were re-divided into 3 subgroups with rapamycin and/or bafilomycin A1 injection.

In experiment 5, 18 C57BL/6J genetically recombined mice were randomly assigned into AAV2/9-hSyn-NC or AAV2/9-hSyn-DNM2 groups with 9 mice in each group treated with rapamycin or 3-methyladenine (3-MA) for 7 days after modeling. All the mice were exposed to 6 km altitude.

All the mice were assigned into the designed groups randomly using a computer-generated allocation sequence. All the experiments were performed and data was analyzed by researchers who were blinded to the mice assignment.

### Hypobaric Hypoxia Model and Drug Administration

The animal HH chamber (Fig. S2) designed by the School of Biomedical Engineering, Fourth Medical Military University was used to simulate high altitude conditions [15]. The housing condition was normalized as a temperature of 26.6 ± 0.4℃ and humidity of 45%–55%. The parameters of different altitudes were set as followings: 4 km 56.0 kPa, 6 km, 47.0 kPa, and 8 km 36.0 kPa.

Pharmacological modulation of autophagic activity was achieved by intraperitoneal injection of rapamycin (Sigma-Aldrich, 553210, 2 mg/kg), bafilomycin A1 (Sigma-Aldrich, 196000, 0.3 mg/kg), and 3-MA (Sigma-Aldrich, 189490, 10 mg/kg) injection according to the previously published articles [16, 17].

### Hematoxylin-Eosin (HE) Staining

After deparaffinization with xylene and rehydration, the brain sections were washed with double-distilled water and stained with hematoxylin for 5–8 min. After washing with water, the sections were then counterstained with eosin for 15–30 s. The color was separated using 1% hydrochloric acid alcohol and controlled under the microscope. Neutral gum was used to seal the sections. Six non-overlapping views in each section of the same cerebral layers were randomly chosen to enumerate the total cell counts and 3 discontinuous sections were chosen for each mouse.

### Fluoro-Jade C (FJ-C) Staining

Injured neurons were stained with FJ-C according to the manufacturer’s protocol for commercial staining kit (TR-100, Biosensis, Australia). Briefly, after deparaffinization, the sections were incubated in 0.06% potassium permanganate solution, followed by incubation in 0.0002% FJ-C solution dissolved in 0.1% acetic acid for 20 min in the dark. Images were photographed with an epifluorescence microscope and analyzed by counting the number of positively stained cells in six non-overlapping views in each section and 3 discontinuous sections for each mouse.

### Construction and Intrahippocampal Injection of Recombination Adeno-associated Viral (AAV) Vectors

The AAV serotype 2/9 vector that expresses the DNM2 coding sequence or scramble control (NC) under neuronal-specific human synapsin (hSyn) core promoter (AAV2/9-hSyn) was and constructed by Genechem CO., Ltd (Shanghai, China). Three-week-old male mGFP-RFP-LC3 or WT mice were weighed and anesthetized with 20 mg/mL ketamine and 5 mL/g 0.4% xylazine. 4 × 10^12^ AAV2/9-hSyn-DNM2 or AAV2/9-hSyn-NC particles were bilaterally infused into the mouse hippocampus area conducted with the stereotaxic surgery according to coordinates as follows: AP = − 2.7 mm, ML = ± 2.7 mm, DV = 2.4 mm. All infusions were made at a rate of 0.1 μL/min by pressure ejection with a 5 μL Hamilton syringe mounted on an infusion pump. Mice were single-housed after the surgery and the subsequent experiments were conducted 2 weeks later.

### Morris Water Maze (MWM) Test

The Morris water maze (MWM) test was conducted to assess hippocampal‐dependent spatial learning and spatial memory as described previously [18, 19]. In brief, the water maze consisted of a circular pool (150 cm diameter, 60 cm depth, 22 ± 2 $^\circ$C) filled with 40 cm of water dyed with nontoxic white paint. The pool was divided into four equal quadrants with spatial clues of each quadrant placed above the water. Mice were trained with four trials per day for 6 days to find a hidden platform 1.5 cm below the water surface (10 cm diameter). During each trial, mice were randomly placed from one quadrant facing the pool well and allowed to swim freely for 60 s. The test ended when mice reached the platform, mice were allowed to stand on the platform for 20 s. If the mice did not find the platform within 60 s, they were guided onto the platform and stayed for 30 s. To assess spatial learning, latency, and path length to the escape platform were measured, and swimming speed was included for completeness. On the final day, mice were placed in the pool for 60 s when the platform was removed to test the reference memory. The congenitally dull mice were excluded according to the reference tests and the enrolled mice were randomly divided into different groups. The behavioral performance had no statistical difference before experimental exposure. The reference memory of mice after exposure to HH was tested at certain time points according to the experimental design. The EthoVision Observer software was used to record the behavior of mice during testing.

### Immunohistochemical (IHC) Staining

IHC staining was performed with Metal Enhanced DAB Substrate Kit (DA1016, Solarbio, China) following the manufacturer’s instructions. Briefly, mice were anesthetized and perfused with 4% paraformaldehyde (PFA). Whole mouse brains were removed and fixed in the same fixative before being embedded in paraffin and cut into 2-μm-thick slices. After deparaffinization, the sections were submerged in EDTA for 20 min at 95 ℃ to retrieve the antigens. Afterward, sections were permeabilized in a blocking solution containing 1% bovine serum albumin (BSA, A1933, Sigma-Aldrich, US) and 0.5% Triton X-100 at room temperature (RT) for 2 h. The sections were then incubated with a primary antibody against DNM2 (Invitrogen, PA1-661, 1:100) dissolved in PBST at 4 ℃ overnight. After rinsing 3 times with PBST, the sections were incubated with a secondary antibody for 2 h at RT and DAB working solution for 30 min at RT in the dark. Images were captured and six non-overlapping views in each section of the same layers were chosen to count the total cells and 3 discontinuous sections were chosen for each mouse.

### Western Blot

Western blotting was performed to analyze the protein expression of Bcl-2 (Abcam, ab16904, 1:1000), Bax (Abcam, ab32503, 1:1000), p-mTOR (Cell Signaling Technology, 5536S, 1:1000), LC3B (Abcam, ab192890, 1:500), P62 (Abcam, ab51416, 1:800), DNM2 (Invitrogen, PA1-661, 1:800) and β-actin (Abcam, ab6276, 1:5000) following protein concentration measurement using the BCA protein assay reagent (Thermo Fisher Scientific, 71285). Proteins (30 μg) were separated by sodium dodecyl sulfate-polyacrylamide gel electrophoresis (SDS-PAGE) and transferred to polyvinylidene difluoride (PVDF) membranes. The membranes were then blocked with 10% no-fat milk fat at room temperature for 1 h before incubation with primary antibodies at 4℃ overnight. Afterward, the membranes were rinsed and incubated with secondary antibodies for 2 h at RT. Bands were then detected by a super signal chemiluminescence detection kit (Sigma-Aldrich, S0500) and images were obtained with the ChemiDoc Imaging System (Bio-Rad, US). Band intensities were quantitated by Image Pro Plus 6.0 software and protein expression was normalized to that of β-actin.

### Transmission Electron Microscopy (TEM)

The morphology of autophagic microstructures in neurons was observed with TEM. Mice were perfused with 4% PFA and the hippocampus was removed and cut into a 1-mm^3^ cube. Tissues were fixed in freshly prepared fixative containing 2.5% glutaraldehyde and 1% paraformaldehyde at RT for 4 h before post-fixed with 0.1% OsO_4_ for 2 h. 70-nm-thick ultrathin sections were stained with 0.2% lead citrate and 1% uranyl acetate. Images were obtained with TEM (HT7800, Hitachi, Japan) at 70 kV.

### Oxidative Stress Measurement

The content of ROS (Elabscience, E-BC-K138-F, China), malondialdehyde (MDA, Elabscience, E-BC-K027-M, E-BC-K028-M, China), superoxide dismutase (SOD, Elabscience, E-BC-K019-M, China), and glutathione (GSH, Elabscience, E-BC-K030-M, China) in hippocampal tissue were measured with commercial kits to indicate oxidative stress in mouse brains as previously described [20]. Briefly, freshly harvested hippocampal tissue was homogenized in a buffer before being centrifuged according to the manufacturer’s protocol. The supernatant was then collected and incubated with detection reagents under specific conditions. The absorbance was detected at 488–535/610 nm (excitation/emission) for ROS, 450, 532, and 600 nm for MDA, 560 nm for SOD, and 412 nm for GSH. The concentrations were calculated and normalized according to standard curves.

### Data Independent Acquisition (DIA) Proteomic Analysis

Each peptide sample was analyzed by liquid chromatography-tandem mass spectrometry (LC-MS/MS) operating in DIA mode by Shanghai Applied Protein Technology Co., Ltd. Each DIA cycle contained one full MS–SIM scan and 30 DIA scans that covered a mass range of 350–1650m/z with the following settings: SIM full scan resolution of 60,000 at 200 m/z; AGC 3e6; maximum IT 50 ms; profile mode; DIA scans were set at a resolution of 30,000; AGC target 3e6; Max IT auto; and the normalized collision energy was 30eV. The runtime was 120 min with a linear gradient of buffer B (80% acetonitrile and 0.1% Formic acid) at a flow rate of 250 nL/min. QC samples (pooled samples from equal aliquots of each sample in the experiment) were injected in DIA mode at the beginning of the MS study and after every 5 injections throughout the experiment, which was used to monitor the MS performance. DIA data were analyzed with Spectronaut Pulsar XTM searching the spectral library constructed as above. The main software parameters were set as follows: the retention time prediction type was dynamic iRT, interference on MS2 level correction was enabled, and cross-run normalization was enabled. All results were filtered based on a Q value cut-off of 0.01 (equivalent to a false discovery rate <1%).

### Statistical Analysis

All results are expressed as the mean ± SD. The analysis was conducted using GraphPad Prism 8 software. Statistical analyses were applied using either Student’s unpaired *t*-test between two groups, one-way ANOVA with Dunnett’s *post hoc* test, or two-ANOVA with Tukey’s *post hoc* test for three or more groups. In the analysis of behavioral tests, two-way ANOVA was applied followed by Bonferroni’s test. Differences with a *P*-value < 0.05 were considered statistically significant. Power analysis was applied using PASS 15 software (NCSS, USA).

## Results

### Acute Exposure to Hypobaric Hypoxia Impairs Cognitive Function in Mice

After exposure to the HH environment, the body weight of mice decreased as altitude and time increased (Fig. S3A). The mortality rate increased with the ascending altitude, and when it increased to ~8 km altitude, >50% of mice were dead by day 7 (Fig. S3B).

**Fig. 1:**
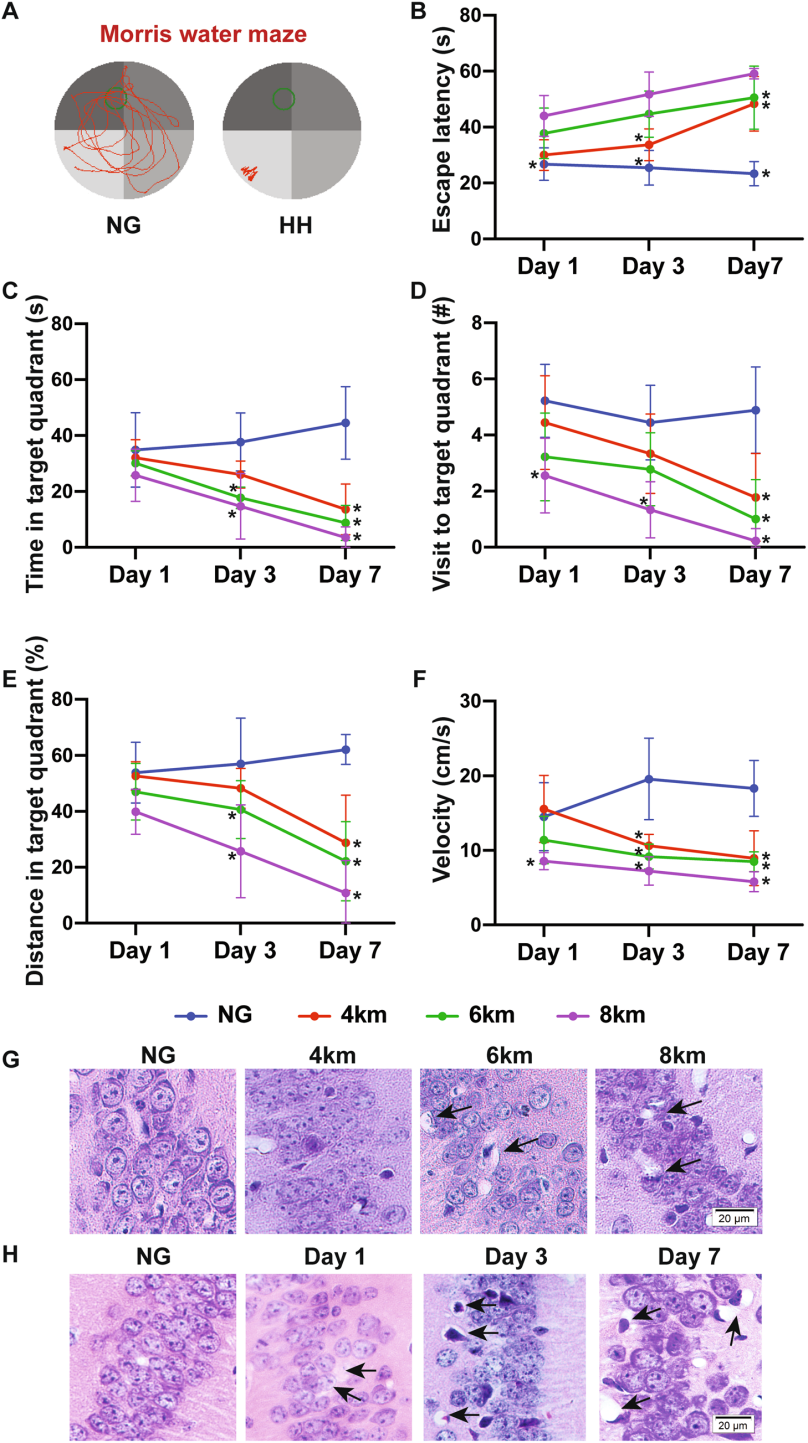
Cognitive function and hippocampal morphology of mice after acute exposure to hypobaric hypoxia (HH). **A** Representative images of the Morris water maze test of mice in the normoxia (NG) and HH groups. **B–F** Escape latency (**B**), time spent in the target quadrant (**C**), the number of mice visiting the target quadrant (**D**), percentage of distance mice spent in the target quadrant (**E**), and mean swim velocity (**F**) on days 1, 3, and 7 in the NG and HH groups at 4, 6, and 8 km altitude, *n* = 9. **G** Representative H&E staining images of neurons in the hippocampus of mice in the NG, 4 km, 6 km, and 8 km altitude groups at 7 days. **H** H&E staining of the hippocampus of mice in a 6 km altitude group exposed at days 1, 3, and 7. The black arrows show neuronal swelling. Scale bars, 20 µm. Values are mean ± SD, *P <0.05 *vs* NG (two-way ANOVA followed by *post hoc* Tukey’s test for multiple comparisons).

The MWM test was used to determine spatial memory after HH exposure. Compared with the mice in the NG group, the mice in the HH groups had varying degrees of memory impairment, which was more severe with ascending altitude and longer time (Fig. 1A–F, Table S1). On day 1, mice in the 8 km HH group had a longer escape latency and slower velocity than those of mice in the NG group while mice in the 4 and 6 km altitude groups had no significant difference in cognitive performance. On days 3 and 7, the visit time and distance in the target quadrant both decreased in all the HH groups except for the 4 km altitude group on day 3. Notably, the mice in the 6 km altitude group had no difference in the number of visits to the target quadrant on day 3 but showed decreases in time and distance in the target quadrant. A distorted hippocampal structure was observed with HE staining which indicated that neurons in the hippocampus were more swollen and sparser than those of mice in the NG group and the nuclei were shrunken in the HH groups on day 7 (Fig. 1G). In addition, there was significant cell loss with the extension of exposure time at 6 km altitude (Fig. 1H). Considering the high mortality rate and evident neurological injury, we chose an altitude of 6 km for subsequent studies.

### Acute Exposure to 6 km Altitude Induces Oxidative Stress and Neuronal Apoptosis in Mouse Hippocampus

**Fig. 2:**
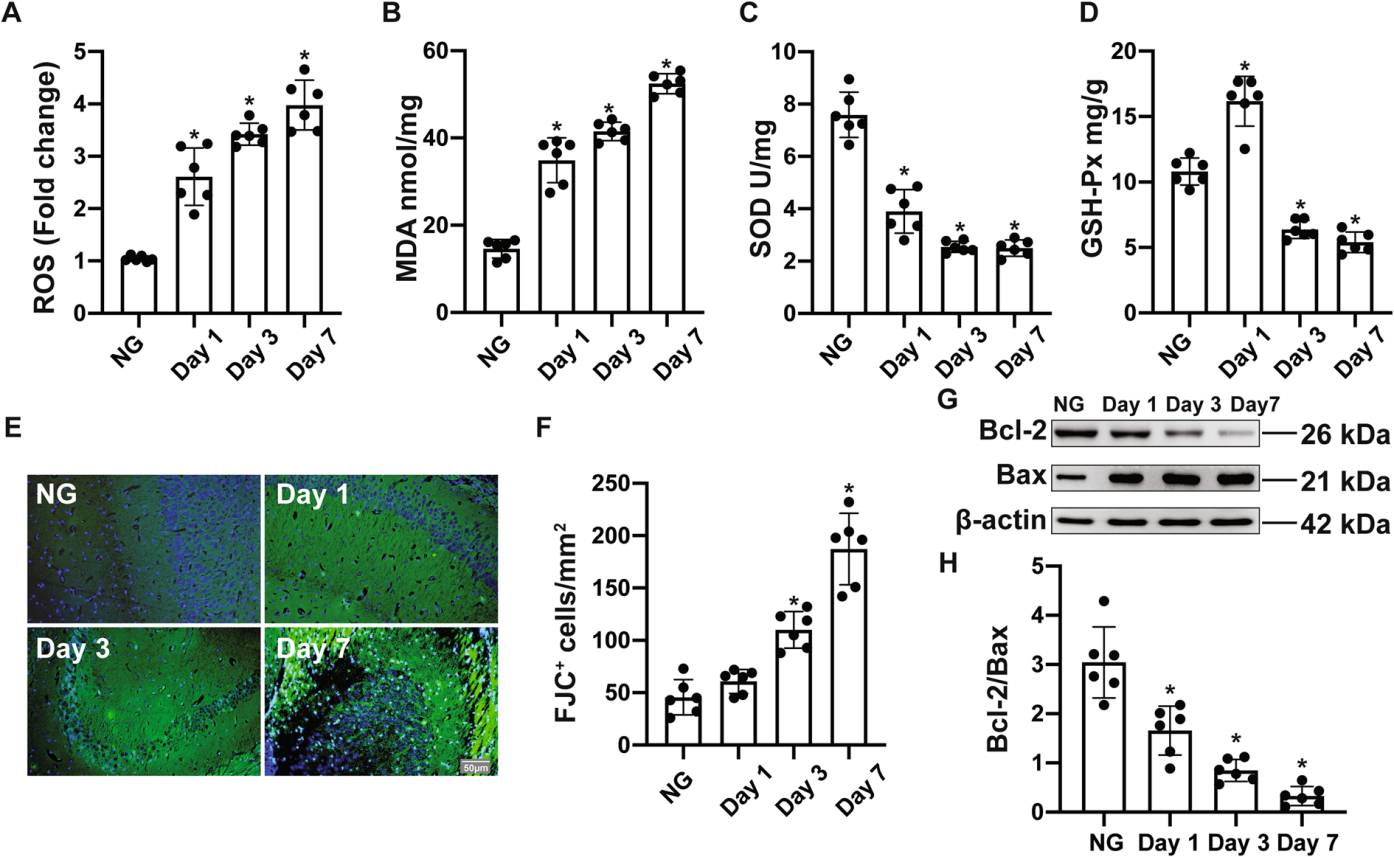
Oxidative stress and neuronal apoptosis in the hippocampus of mice exposed to 6 km altitude. **A**–**D** C57BL/6J mice were exposed to 6 km altitude and sacrificed on days 1, 3, and 7 and the hippocampus was harvested for the study of the production of ROS (**A**), which is expressed as fold-change over the NG group, the levels of MDA (**B**), the activity of SOD (**C**), and the levels of GSH-Px (**D**). **E** Fluoro-jade staining (FJC, green) staining showing degenerated neurons in the hippocampus of the NG, day 1, day 3, and day 7 groups. Cell nuclei are stained with DAPI (blue). Scale bar, 50 µm. **F** The numbers of JC-1-positive cells in each group. **G** Western blots and analysis of Bcl-2 and Bax protein expression in mouse hippocampus; β-actin was used as an internal standard. **H** The bands were quantified using ImageJ and the density histograms for Bcl-2/Bax used GraphPad Prism 8. Values are the mean ± SD, *n* = 6. *P <0.05 *vs* the NG group (one-way ANOVA followed by *post hoc* Tukey’s test for multiple comparisons).

To evaluate the oxidative stress in HH, we measured the production of ROS and concentrations of antioxidant enzymes in mice brains. Compared with those of the NG group, the ROS and MDA concentrations significantly increased in a time-dependent manner (Fig. 2A, B, Table S2). The antioxidant enzyme SOD decreased on days 1, 3, and 7 when mice were exposed to HH (Fig. 2C, Table S2). Intriguingly, GSH-Px was significantly increased on day 1 when exposed to 6 km altitude but decreased on days 3 and 7 (Fig. 2D, Table S2). Furthermore, we used Fluoro-jade staining to detect neurodegeneration in the hippocampus. The number of degenerated neurons was significantly augmented on days 3 and 7 after exposure to 6 km altitude compared with that of the NG group (Fig. 2E, F, Table S3). The significantly increased ratio of Bcl-2 to Bax revealed that HH resulted in cellular apoptosis in the hippocampus (Fig. 2G, H, Table S4).

### Data Independent Acquisition Mass Spectrometry of the Proteomic Profiles of Hippocampal Protein in Mice Brain Under Hypobaric Hypoxic Conditions

**Fig. 3:**
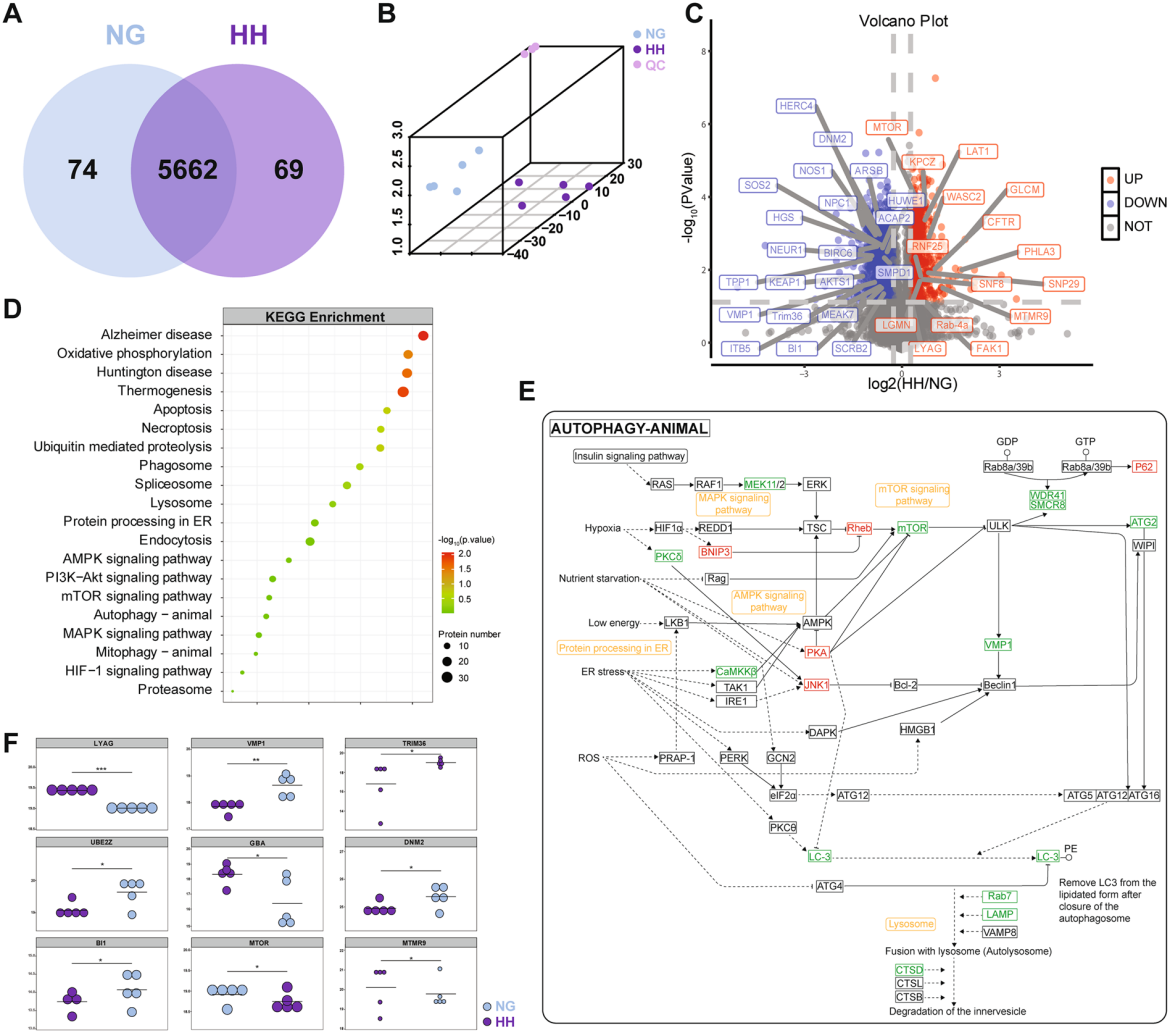
Data-independent acquisition mass spectrometry of the proteomic profiles of hippocampal protein in mouse brains under hypobaric hypoxic. The hippocampus of the NG and HH (6 km altitude, 7 days) groups (NG and HH) subjected to mass spectrometry assay for data-independent acquisition (DIA)-based proteomic analysis (*n* = 5). **A** Overlap of proteins between the NG and HH groups was detected with DIA. **B** Orthogonal Projections to Latent Structure Discriminant Analysis (OPLS-DA) was used to provide insights into the separation between NG and HH groups. **C** Volcano plot of differentially-regulated proteins (DEPs; red up, blue down) in NG and HH groups. **D** Kyoto Encyclopedia of Genes and Genomes (KEGG) analysis revealed enriched pathways related to acute hypobaric hypoxic brain damage. **E** The DEPs (red up, green down) and enriched pathways (yellow) in the autophagy-animal pathway map according to KEGG. **F** The expression of autophagy-related proteins such as LYAG, VMP1, TRIM36, UBE2Z, GBA, DNM2, BI1, MTOR, and MTMR9 in the NG and HH groups.

To further analyze proteomic changes under a plateau environment, we applied a mass spectrometry assay for DIA-based proteomic analysis on 5 mouse hippocampi each from the NG and HH (6 km altitude, 7 days) groups. A total of 93055 distinct peptides in an assembly of 5808 proteins were identified with a protein-level false discovery rate of 0.01 (Fig. 3A). Orthogonal Projections to Latent Structure Discriminant Analysis (OPLS-DA) were used to provide insights into the separation between the HH and NG groups (Fig. 3B). We identified 1385 differentially-expressed proteins (DEPs) at *P* <0.05 with a fold-change >1.2 or <0.83, among which 381 proteins were up-regulated and 1004 were down-regulated in the HH group compared with those of the NG group (Figs. 3C, S4). Kyoto Encyclopedia of Genes and Genomes (KEGG) pathway enrichment analysis revealed that multiple pathways were involved in acute hypobaric hypoxic brain damage, such as oxidative phosphorylation, ubiquitin-mediated proteolysis, lysosomes, autophagy, and the mTOR signaling pathway (Fig. 3D). We further screened out 16 DEPs enriched in the autophagy pathway map and 5 related pathway terms, such as BNIP3, mTOR, P62, and LAMP (Fig. 3E). Combined with the Uniprot database, the lysosomal proteins LYAG and GBA were upregulated, and proteins inhibiting autophagy (VMP1) decreased, while MTOR (mechanistic target of rapamycin kinase). which inhibits autophagy, increased in the HH group compared with the NG group (Fig. 3F).

### Hypobaric Hypoxia Inhibits Autophagic Activity in Neurons

**Fig. 4:**
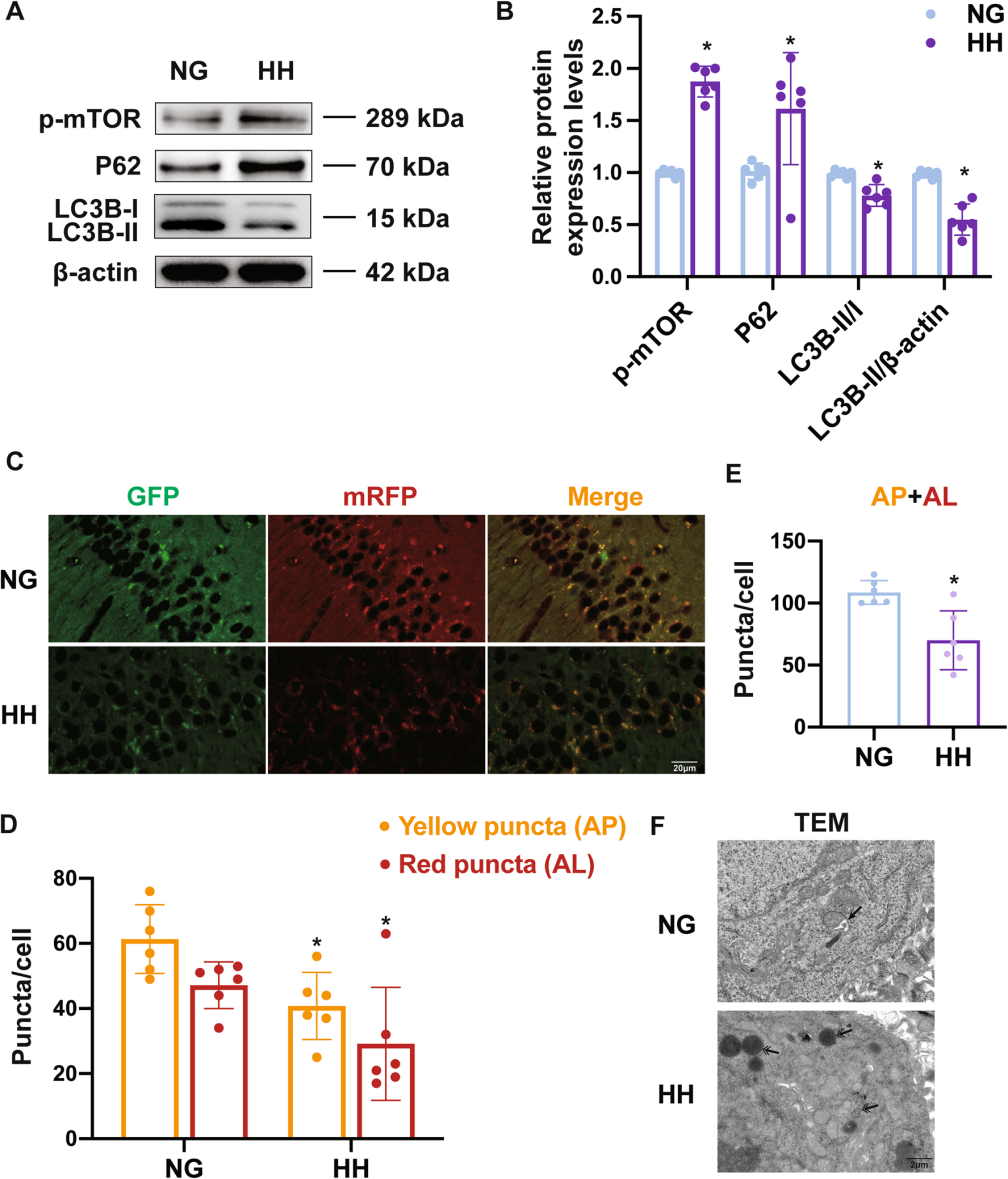
Autophagic activity in mouse hippocampus after hypobaric hypoxia. **A** Western blot was used to measure the expression of autophagy-related proteins (mTOR, P62, LC3B-I, LC3B-II), and β-actin. **B** Density histograms of protein relative to β-actin. **C** Tandem mGFP-RFP-LC3 transgenic mice were used to monitor the autophagic flux in the hippocampus. Autolysosomes are labeled as red puncta, and autophagosomes are labeled as yellow puncta. Scale bars, 20 µm. **D, E** Numbers of degraded autolysosomes (red puncta) and undegraded autophagosomes (yellow puncta) in **C**. **F** Transmission electron microscopy images showing the structure of autophagic organelles. The black arrows indicated autolysosomes and the double arrows indicate autophagosomes or lysosomes. Scale bar, 2 µm. Data are presented as the mean ± SD, *n* = 6, *P <0.05 *vs* NG group (one-way ANOVA followed by *post hoc* Tukey’s test for multiple comparisons, and Student’s *t*-test between two groups).

To verify the results of DIA and explore the role of autophagic activity in HH-induced brain damage, the protein levels of the autophagic markers mTOR, P62, and LC3B in the hippocampus of mice exposed to 6 km for 7 days were measured by Western blotting. HH resulted in a significant increase in the mTOR and P62 expression levels and a decrease in the LC3B-II/-I ratio and LC3B-II expression (Fig. 4A, B, Table S5). The presence of LC3B in neurons was confirmed by double-immunofluorescence staining with a neuron marker and HH reduced the number of LC3B puncta in neurons (Fig. S5). Next, autophagic flux was measured in tandem mRFP-GFP-LC3B transgenic mice. As expected, fewer autophagosomes (APs) and autolysosomes (ALs) were found in the mouse hippocampus when exposed to HH (Fig. 4C–D, Table S5). To confirm the findings transmission electron microscopy (TEM) was applied to monitor autophagic activity. In neurons of the hippocampus in the HH group, the formation of ALs was inhibited compared with the NG group (Fig. 4E). Together, these results indicated that autophagic flux is reduced in neurons after acute HH exposure.

### Decrease of DNM2 Contributes to Autophagic Flux Impairment in Neurons After Hypobaric Hypoxia

**Fig. 5:**
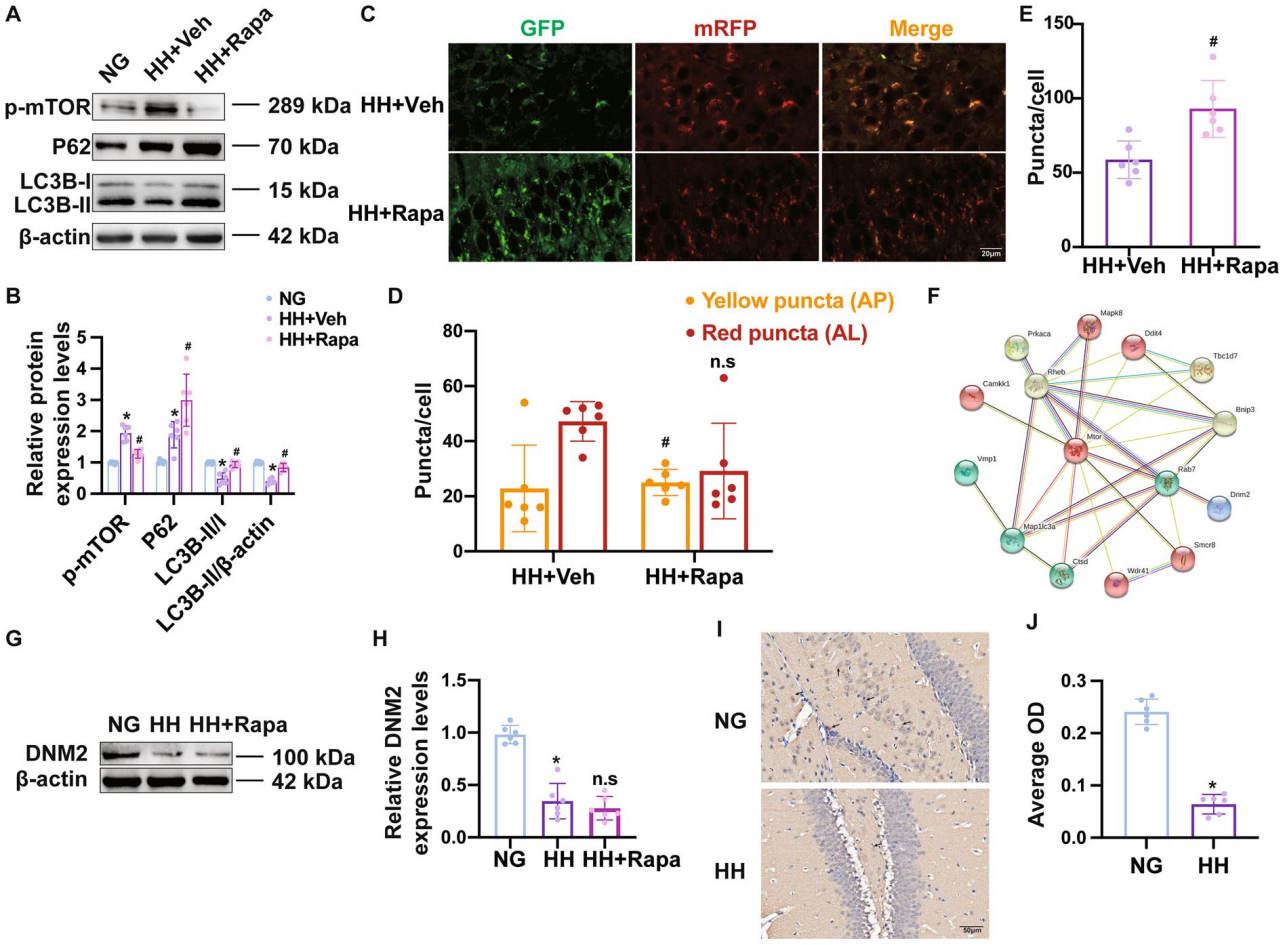
Decrease of DNM2 blocks autophagic flux in hippocampal neurons after hypobaric hypoxia. **A** Western blot of the expression of mTOR, P62, LC3B-I, and LC3B-II protein in the hippocampus. β-actin was used as an internal standard. **B** Relative protein expression levels, *n* = 6. **C** Fluorescent images from mGFP-RFP-LC3 mice treated with rapamycin. Autolysosomes are labeled as red puncta, and autophagosomes as yellow puncta. Scale bar, 20 µm. **D, E** Numbers of autolysosomes (red puncta) and undegraded autophagosomes (yellow puncta), *n* = 6. **F** The STRING database indicates that RAS-related GTP-binding protein 7 (Rab7) has close connections with multiple DEPs including DNM2 which may play a key role in the autophagic activity. **G, H** Western blot used to measure the expression of DNM2 in the mouse hippocampus (**G**) and relative integrated density histograms of DNM2 protein to β-actin (**H**), *n* = 6. **I, J** Representative images of immunohistochemical staining (**I**) and the average optical density (**J**) of DNM2 in the hippocampus (*n* = 6), scale bar, 50 µm. Data are presented as the mean ± SD, *P <0.05 *vs* NG group and ^#^*P* <0.05 *vs* HH+Veh group (one-way ANOVA followed by *post hoc* Tukey’s test for multiple comparisons, and Student’s *t*-test between two groups).

Since autophagy was inhibited in neurons, we next used rapamycin, a specific pharmacological inhibitor of mTOR, to initiate autophagy [21]. The expression of mTOR decreased, and the LC3B-II/-I ratio and LC3B-II expression increased in the hippocampus of mice treated with rapamycin (Fig. 5A, B, Table S7). The expression of LC3B in neurons treated with rapamycin was confirmed by double-immunofluorescence staining (Fig. S5). We noted that P62 expression increased with rapamycin treatment (Fig. 5A, B). It has been reported that the accumulation of P62 indicates the inhibition of autophagic flux [9]. Consistent with that, we observed more APs while the AL number was not significantly changed (Fig. 5C–E, Table S8).

To explore the potential molecular signaling in regulating downstream autophagic activity, we reviewed the DIA results and noted that, among the DEPs in the autophagy pathway, RAS-related GTP-binding protein 7 (Rab7) had close connections with multiple DEPs according to the STRING database (Fig. 5F). Rab7 is a well-characterized AP sorting protein and has been shown to enhance the formation of ALs in multiple cells such as hepatic cells and neurons [22]. We found that dynamin-2 (DNM2) may be associated with Rab7 and play a significant role in autophagic activity. In agreement with the DIA results, Western blot analysis confirmed that DNM2 expression significantly declined in the HH group, but it did not change with rapamycin treatment (Fig. 5G–J, Table S7). The decreased expression of DNM2 in hippocampal neurons after HH exposure was confirmed with immunofluorescence (Fig. S6).

### Overexpression of Neuronal DNM2 Enhances Autophagic Flux in Hippocampal Neurons After Hypobaric Hypoxia Injury

**Fig. 6:**
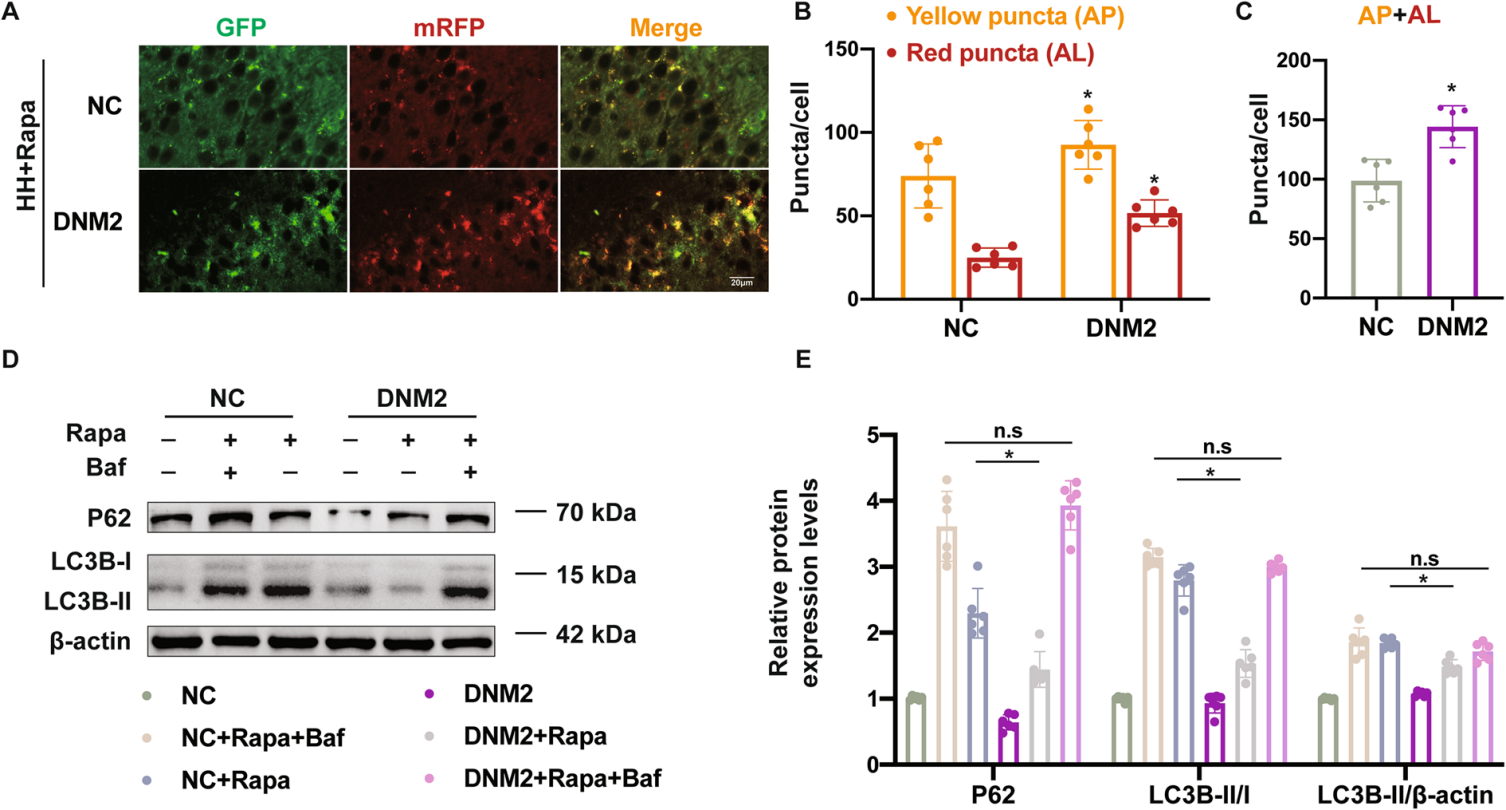
Autophagic flux after DNM2 overexpression in combination with rapamycin treatment. **A** mGFP-RFP-LC3 mice received AAV2/9-hSyn-NC or AAV2/9-hSyn-DNM2 transfection (NC and DNM2) before rapamycin treatment. Fluorescent images of mGFP-RFP-LC3 hippocampus indicate autophagic flux in the NC and DNM2 groups. Autolysosomes are labeled as red puncta, and autophagosomes as yellow puncta. Scale bar, 20 µm. **B** Numbers of degraded autolysosomes (red puncta) and undegraded autophagosomes (yellow puncta). **C** Total numbers of yellow and red puncta in the NC and DNM2 groups. **D** Mice received rapamycin and/or bafilomycin A1 injection with or without DNM2 overexpression before the hippocampus was removed and homogenized. Western blots were used to detect P62, LC3B-I, and LC3B-II protein expression. **E** Relative integrated density of proteins is depicted in the histograms, and β-actin was used as the loading control. Data are presented as the mean ± SD, *n* = 6, *P <0.05 *vs* the NG group, and ^#^*P* <0.05 *vs* HH+Veh group (one-way ANOVA followed by *post hoc* Tukey’s test for multiple comparisons and Student‘s *t*-test between two groups).

Recent research has shown that DNM2 directly binds to LC3B and is critical for AP-lysosome fusion [23]. To explore its role in HH brain damage, AAV2/9-hSyn-DNM2 was applied to genetically upregulate the expression of neuronal DNM2 in both tandem mRFP-GFP-LC3B transgenic and WT mice combined with rapamycin treatment (Fig. S7A, B). Compared with the AAV2/9 vector, DNM2 overexpression enhanced the formation of APs and ALs (Fig. 6A–C, Table S8). To further investigate the role of DNM2 in autophagic flux, mice were pretreated with a lysosomal inhibitor, bafilomycin A1 (Baf). Compared with the AAV2/9-NC group, DNM2 overexpression reduced the LC3B-II/-I ratio as well as LC3B-II and P62 expression when treated with rapamycin but not in combination with Baf (Fig. 6D, E, Table S9), suggesting that DNM2 regulates a step downstream of AP and lysosome fusion. Taken together, the above results indicated that DNM2 is required for the maintenance of autophagic flux. In combination with rapamycin treatment, DNM2 overexpression promotes autophagic flux in neurons after HH.

### Enhanced Autophagy Alleviates Oxidative Stress and Neuronal Loss After Hypobaric Hypoxia

**Fig. 7:**
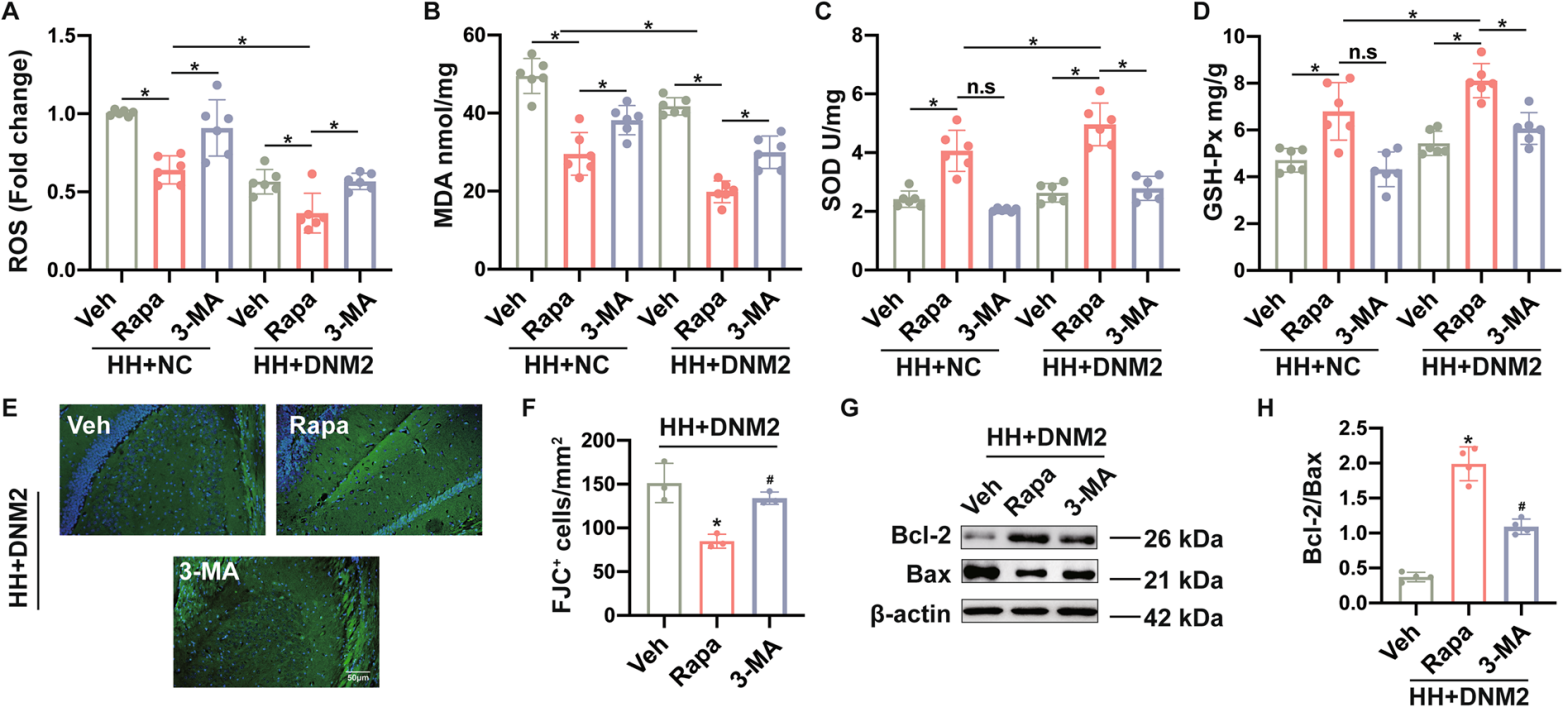
Oxidative stress and neuronal loss after autophagic flux is enhanced in hypobaric hypoxia. The mice received rapamycin (Rapa) injections or 3-methyladenine (3-MA) in the condition of DNM2 overexpression or negative control AAV2/9. For control, the mice received vehicle injection (Veh). All the above mice were sacrificed on day 7 after hypobaric hypoxia. **A**, **B** The expression of the oxidative stress products ROS (**A**) and MDA (**B**). **C**, **D** The anti-oxidative enzymes SOD (**C**) and GSH (**D**) as also measured by commercial reagent kits. **E** FJ-C staining (green) indicates degenerating hippocampus neurons after hypobaric hypoxia with DNM2 overexpression. Cell nuclei are stained with DAPI (blue). **F** Numbers of JC-1 positive cells in the hippocampus of the Veh, Rapa, and 3-MA groups were calculated using Image Pro Plus 6.0. **G** Western blots were used to measure Bcl-2 and Bax protein expression in the hippocampus after hypobaric hypoxia with DNM2 overexpression. β-actin was used as an internal standard. **H** analysis of Bcl-2/Bax. Data are presented as the mean ± SD, *n* = 6. * in **A**–**D** indicates *P* <0.05 and n.s. refers to no statistical difference. *P <0.05 in **F** and **H** *vs* the Veh group, and ^*#*^*P* <0.05 *vs* the Rapa group (one-way ANOVA followed by *post hoc* Tukey’s test for multiple comparisons).

To study the contribution of autophagy to HH-ischemic brain injury, we used autophagy inhibitor 3-MA to suppress autophagic flux. When mice were treated with rapamycin after exposure to 6 km altitude, the ROS and MDA concentrations decreased, whereas the SOD and GSH concentrations increased in the hippocampus. The use of 3-MA significantly reduced the anti-oxidative effects of autophagy enhancement and these changes were more evident when DNM2 was overexpressed (Fig. 7A–D, Table S10). Fluoro-jade staining indicated that autophagy enhancement significantly reduced the JC-1^+^ cell number, which was increased by 3-MA treatment (Fig. 7E, F, Table S11). In addition, autophagic flux enhancement significantly increased the Bcl-2 to Bax ratio, which was decreased in the presence of 3-MA (Fig. 7G, H, Table S12). These results suggested that autophagy enhancement has anti-oxidative effects and reduces neuronal loss after HH.

### Enhanced Autophagy Attenuates Cognitive Deficits of Mice After Hypobaric Hypoxia

**Fig. 8:**
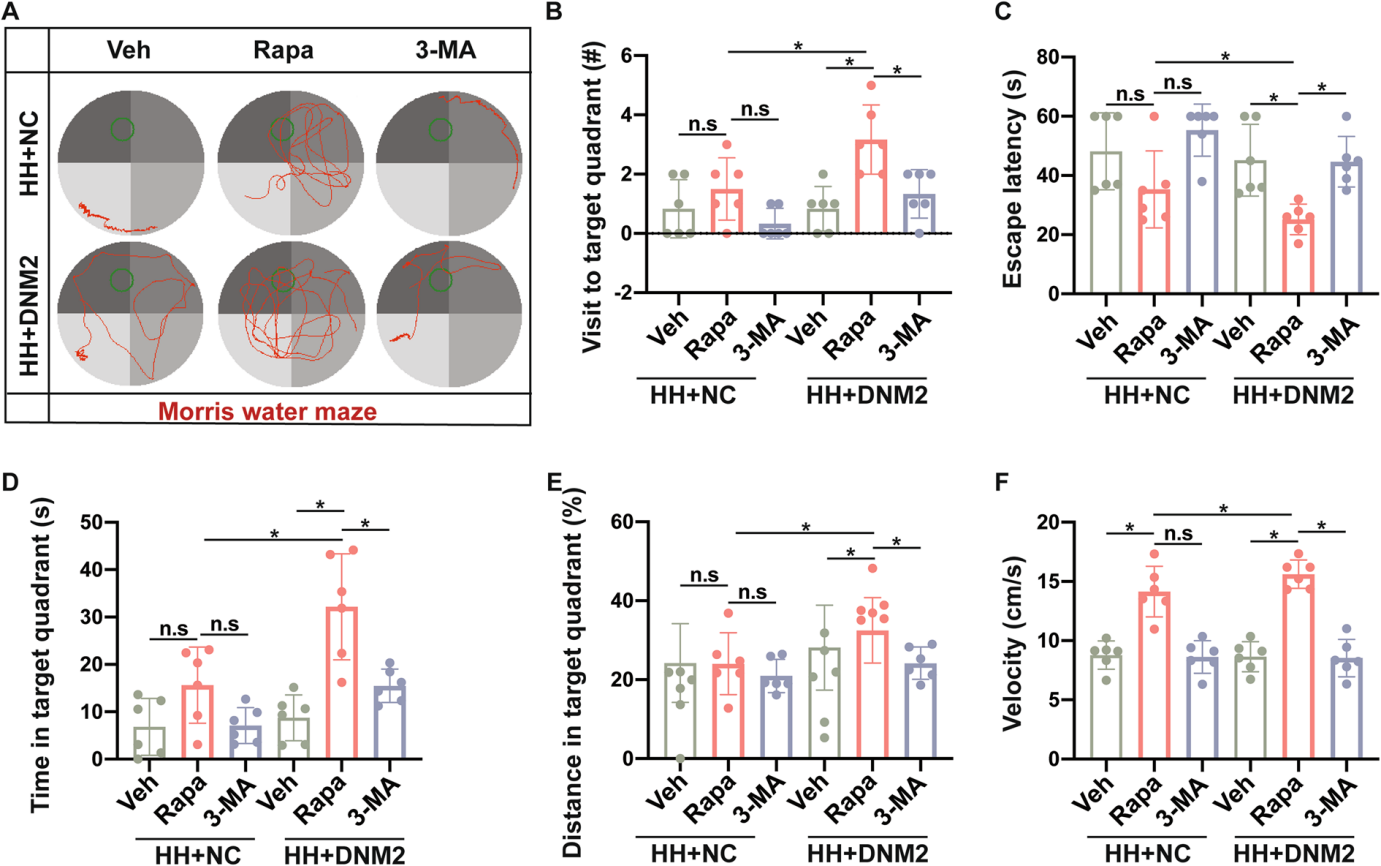
Cognitive deficits of mice after hypobaric hypoxia when the autophagic flux is enhanced. **A** Representative path patterns in the Morris water maze indicate the behavioral performance in each group. **B**-**F** The number of times to target quadrant (**B**), escape latency (**C**), time spent in the target quadrant **D**, percentage of swim distance in the target quadrant (**E**), and mean swim velocity (**F**) measured in the condition of rapamycin or 3-MA with or without DNM2 overexpression. Data are presented as the mean ± SD, *n* = 6, * in **A**–**D** indicates *P* <0.05 and n.s. refers to no statistical difference (one-way ANOVA followed by *post hoc* Tukey’s test for multiple comparisons).

The effect of enhanced autophagy on cognitive function after HH was further analyzed using the MWM. Rapamycin had limited effects on cognitive performance on day 7 of exposure to 6 km altitude but improved the velocity of mice. In the condition of DNM2 overexpression, rapamycin had protective effects against the HH-induced neurological deficits, and this was abolished by 3-MA (Fig. 8A–F, Table S13). The above results indicated that enhancement of autophagic flux has a protective effect on cognitive performance after HH.

## Discussion

In the present study, we demonstrated that HH exposure led to cognitive deficits, structural damage, and oxidative stress on the hippocampal neurons of mice in a time- and altitude-dependent manner. The impairment of autophagic flux was a part of the underlying pathological mechanism. This is a novel study focused on the protective role of autophagy against high-altitude-induced neuronal injury and oxidative stress in a rodent model. Using a proteomic method, we have found that DNM2 may participate in AP-lysosome fusion in neurons and is critical for maintaining autophagic flux.

Rapid ascent to high altitudes can cause AMS, leading to potentially life-threatening HACE. Apart from the >140 million people who reside at HA above 2500 meters, many people travel worldwide to HA locations. Among them, ~30 to 50% develop symptoms of AMS, and 1% to 2% develop HACE [24]. HH is one of the most important environmental factors of HA. It is likely to expose the brain to HH stress as it is highly oxidative. Consistent with previous studies [25], in the present study, we found that spatial memory was impaired in mice performing the MWM test during the time frame examined, and this worsened as the altitude increased to 8 km altitude, except at 4 km altitude. Indeed, 6 km is an extreme altitude that can hardly be reached by humans. One reason may be that rodents are more tolerant to HH than mammals, the other reason may be in the real plateau, there are many other deleterious factors such as cold and ultraviolet light. Morphologically, the neuronal loss in the hippocampus was partially in accordance with HA-induced oxidative stress in cognitive disturbances. In the formation of HACE, both vasogenic and cytotoxic edema might be involved, and other types of cells in the brain may also play an essential role in the process such as vascular endothelial cells and glia [26]. Among them, microglia were activated to release pro-inflammatory cytokines if exposed to deleterious signals including cellular stress, which therefore led to neuronal injury. The microenvironments in the brain are quite complex under HH exposure and the cross effects between multiple cells need further study.

**Fig. 9:**
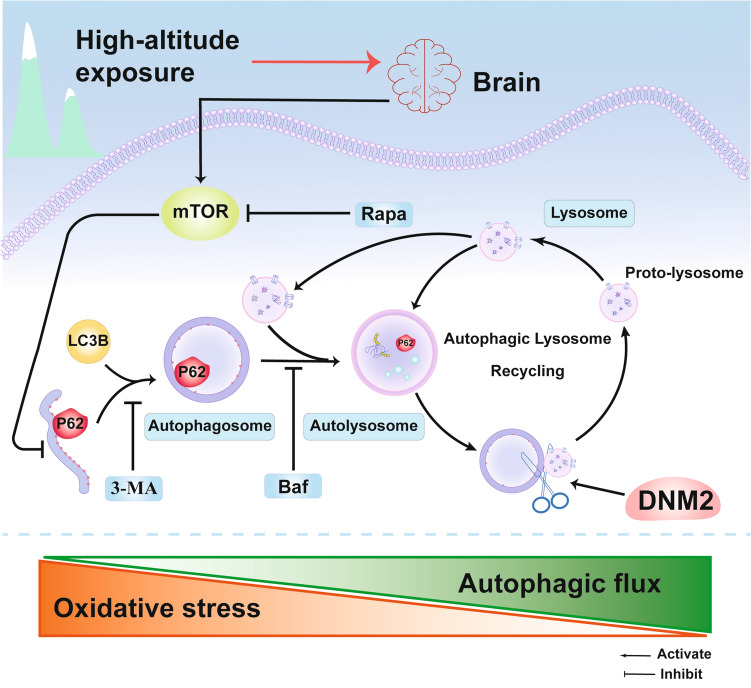
A working model indicating that autophagic flux attenuates oxidative stress after acute hypobaric hypoxia injury. Autophagic flux is impaired after hypobaric hypoxia exposure as indicated by the blockade of both autophagic initiation and autolysosome formation regulated by mTOR and DNM2; respectively. The promotion of autophagic flux contributes to an anti-oxidative system enhancement, which leads to a decrease in ROS production and thereby attenuates brain injury and neuronal loss.

Autophagy is a highly conserved cellular process that exerts a cytoprotective effect by removing damaged organelles [27]. Multiple stimuli such as starvation, amino-acid or glucose deprivation, and low O_2_ tension can trigger autophagy [28, 29]. Among those stressors, hypoxia is a well-known autophagy inducer in various cell types [30]. Interestingly, as one of the most important environmental characteristics of HA, HH inhibits autophagic activity as shown in our study. According to the previously published literature on humans, the physiological response is different between normobaric hypoxia and HH, as indicated by lower peripheral O_2_ saturation (SpO_2_) and higher heart rate (HR) in HH [31], which may partially be the reason for the above difference regarding autophagy. Conventional autophagy is not static but rather a highly dynamic, multi-step process that encompasses the inclusion/exclusion of cargo within the AP, the delivery of cargo to lysosomes, and its subsequent breakdown [32]. To our knowledge, this study is the first to uncover the association between autophagy and HH-related brain injury in mice. In the current study, tandem mRFP-GFP-LC3B transgenic mice were used to assess the autophagic flux. As a well-known upstream regulator of autophagy, mTOR plays an essential role in negatively regulating autophagy [33]. Thus, we further assessed the capacity of autophagy induction using the classic autophagy inducer rapamycin targeting mTOR. Indeed, rapamycin induced a significant increase in the total number of autophagic vacuoles but had a limited impact on the formation of ALs. Therefore, we speculated that autophagic flux is impaired after HH. Through DIA proteomics analysis, we found that the neuronal DNM2 levels decreased after HH, which may contribute to the impairment of autophagic flux. DNM2 overexpression promoted AP and AL formation following treatment with rapamycin. Taken together, these results indicated that in acute exposure to HH, neuronal autophagic flux is disturbed by the impediment of AL formation and a decrease in autophagy induction (Fig. 9).

DNM2 belongs to the dynamin family of large GTPases that mediate membrane fission during multiple cellular processes including endocytosis and organelle division/fusion [34]. Most studies on DNM2 have focused on neuromuscular diseases such as centronuclear myopathies. It is known that DNM2 is a fundamental component of the mitochondrial division machinery [35]. Under hypoxic conditions, dynamic mitochondrial homeostasis is responsible for neuronal survival [36, 37]. In the present study, we found that overexpression of DNM2 significantly suppressed ROS, which causes the deterioration of mitochondrial function apart from causing oxidative stress under HH conditions. However, further studies are still needed to evaluate the exact role of DNM2 in regulating mitochondrial function. Recently, an intensive study showed that DNM2 catalyzes membrane fission during the release of endocytic vesicles [38]. Besides, accumulating evidence suggests that DNM2 plays an important role in regulating autophagic flux in mammalian cells. Schulze and colleagues demonstrated that DNM2 functions as an active mediator of AL reformation in the hepatocyte [39]. Moreover, the direct enhancement of DNM2 activity results in autophagy initiation in chronic myeloid leukemia [38]. The relationship between DNM2 and autophagic flux is underscored by our findings. DNM2 overexpression enhanced AP-lysosome fusion, thus promoting autophagic flux balance and enhancing the effects of rapamycin in mouse brains exposed to HA. The promotion of DNM2-related autophagy also attenuates brain injury and neuronal loss, indicating that DNM2 may serve as a potential therapeutic target for interventions in HH brain injury. However, the precise role of DNM2 in HH brain injury still requires further study, and DNM2 mutant mice should be obtained to confirm the underlying mechanism of its effect on damaged neurons, which is part of our limitations.

Stimulation of autophagy has been considered a double-edged sword in many diseases. It has been reported that blocking autophagic flux during cerebral ischemia/reperfusion aggravates neuronal damage [40]. Here, we used AAV2/9-hSyn-DNM2 in combination with rapamycin to promote neuronal autophagy. We found that enhancement of autophagy attenuated oxidative stress and cognitive function disturbances in the HH situation, while 3-MA, the autophagy inhibitor, abolished the above protective effects. Therefore, these findings support the idea that the impairment of autophagic flux contributes to HH-induced brain injury. Intervention in autophagy may be used as a potentially valuable treatment for the clinical prevention of HA brain damage.

### Supplementary Information

Below is the link to the electronic supplementary material.

Supplementary file1 (PDF 1293 KB)
