# Supplementary material for: Impairment of Autophagic Flux After Hypobaric Hypoxia Potentiates Oxidative Stress and Cognitive Function Disturbances in Mice
Source: Neurosci Bull. 2023 Aug 22;40(1):35–49. doi: 10.1007/s12264-023-01099-6 (PMC10774493; doi:10.1007/s12264-023-01099-6)
Supplement: Supplementary file 1 — Supplementary file1 (PDF 1293 KB) [file 12264_2023_1099_MOESM1_ESM.pdf]

Supplementary Materials

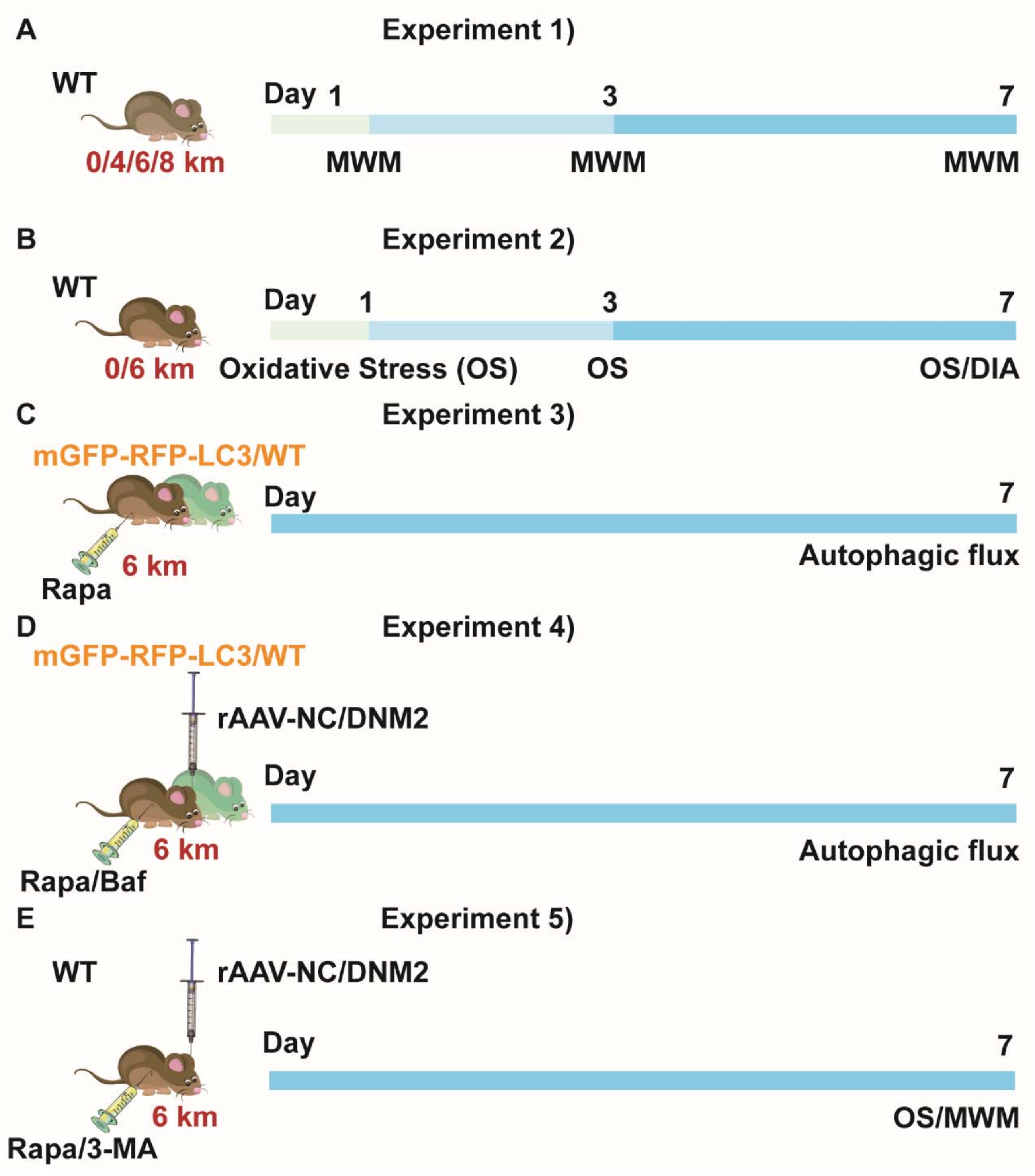

Fig. S1 Schedules of the experimental design and group assignment. **A** C57BL/6J mice (n = 59) were randomly divided into 4 groups: the normoxia group (NG, n = 9) and hypobaric hypoxia (HH) groups

exposed to 4 ($n = 15$), 6 ($n = 15$), and 8 ($n = 20$) km altitude. Behavioral tests were performed on days 1, 3, and 7 of HH exposure. **B** The mice exposed to 6 km were used for the oxidative stress analysis on days 1, 3, and 7, and proteomics analysis was performed on day 7. **C** Tandem mGFP-RFP-LC3 transgenic mice ($n = 6$) and C57BL/6J mice ($n = 24$) were exposed to 6 km altitude for 7 days and 6 mGFP-RFP-LC3 mice were assigned to the NG group. C57BL/6J mice were divided into two groups each receiving blank solvent (HH+Veh) or rapamycin (HH+Rapa). All samples were used for the autophagic flux text. **D** mGFP-RFP-LC3 mice ($n = 12$) were divided into two groups and received AAV2/9-hSyn-NC or AAV2/9-hSyn-DNM2 transfection before hypobaric hypoxia exposure. C57BL/6J mice ($n = 54$) were divided into two groups that received AAV2/9-hSyn-NC or AAV2/9-hSyn-DNM2 transfection and in each group, 27 mice were divided into 3 subgroups that received rapamycin and/or bafilomycin A1 injection to measure the autophagic flux. **E** C57BL/6J genetically recombined mice ($n = 18$) were randomly and evenly assigned into AAV2/9-hSyn-NC or AAV2/9-hSyn-DNM2 groups. In each group, mice were injected with blank solvent, rapamycin, or 3-methyladenine (3-MA). All the mice were treated under 6 km hypobaric hypoxia exposure for 7 days.

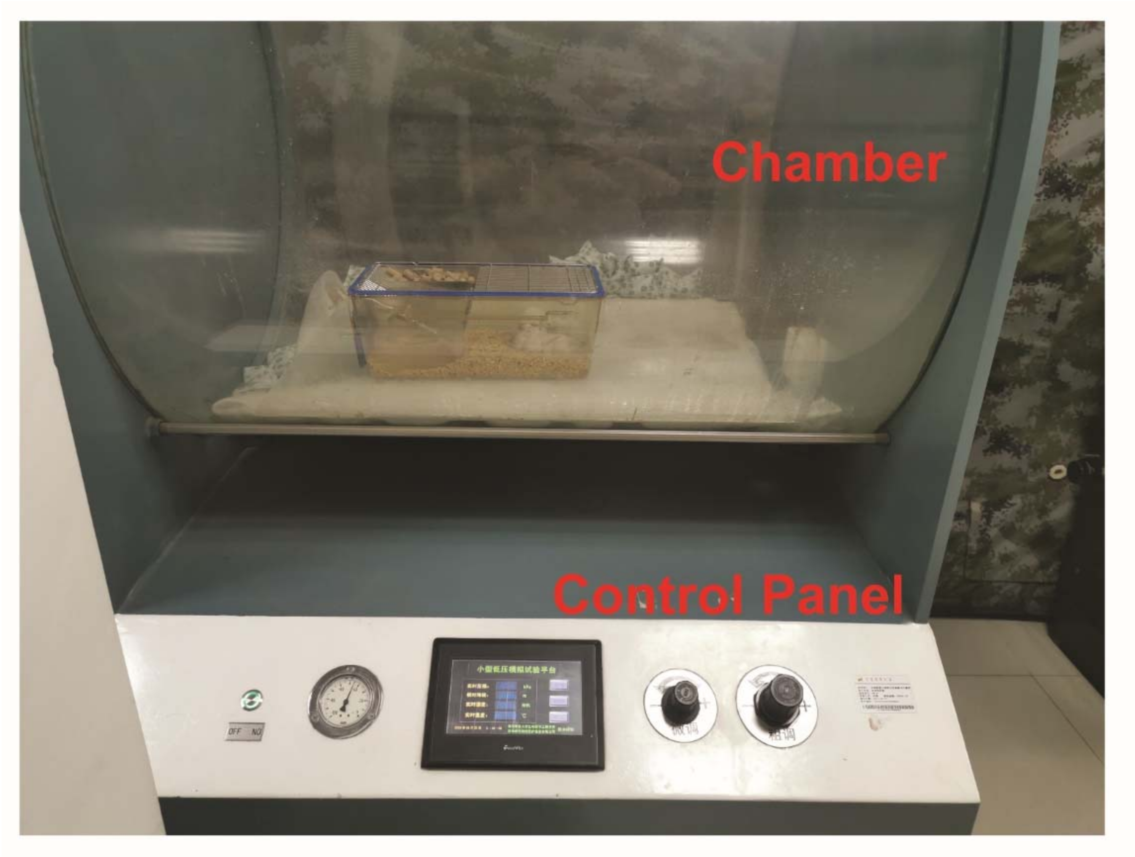

Fig. S2 The hypobaric hypoxia chamber used in the present study.

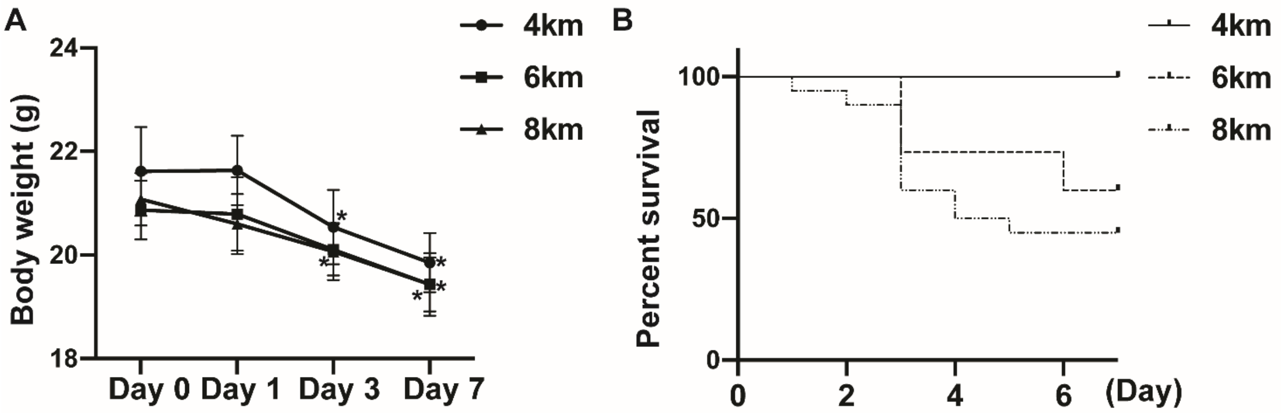

Fig. S3 The health condition of mice after hypobaric hypoxia. **A** The body weight of mice exposed to hypobaric hypoxia. **B** Survival analysis of mice exposed to HH.

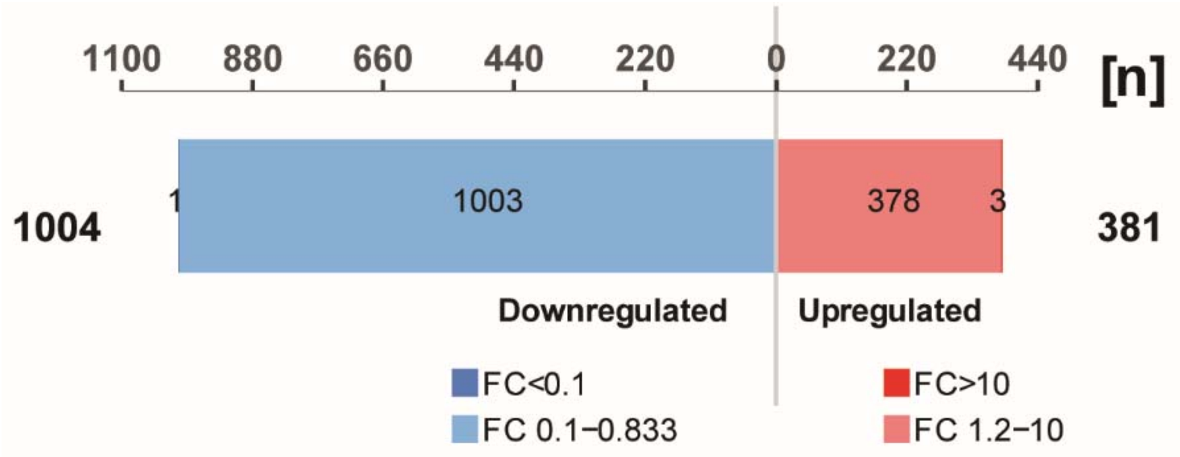

Fig. S4 The numbers of differentially-expressed proteins in the hypobaric hypoxia group compared with the NG group.

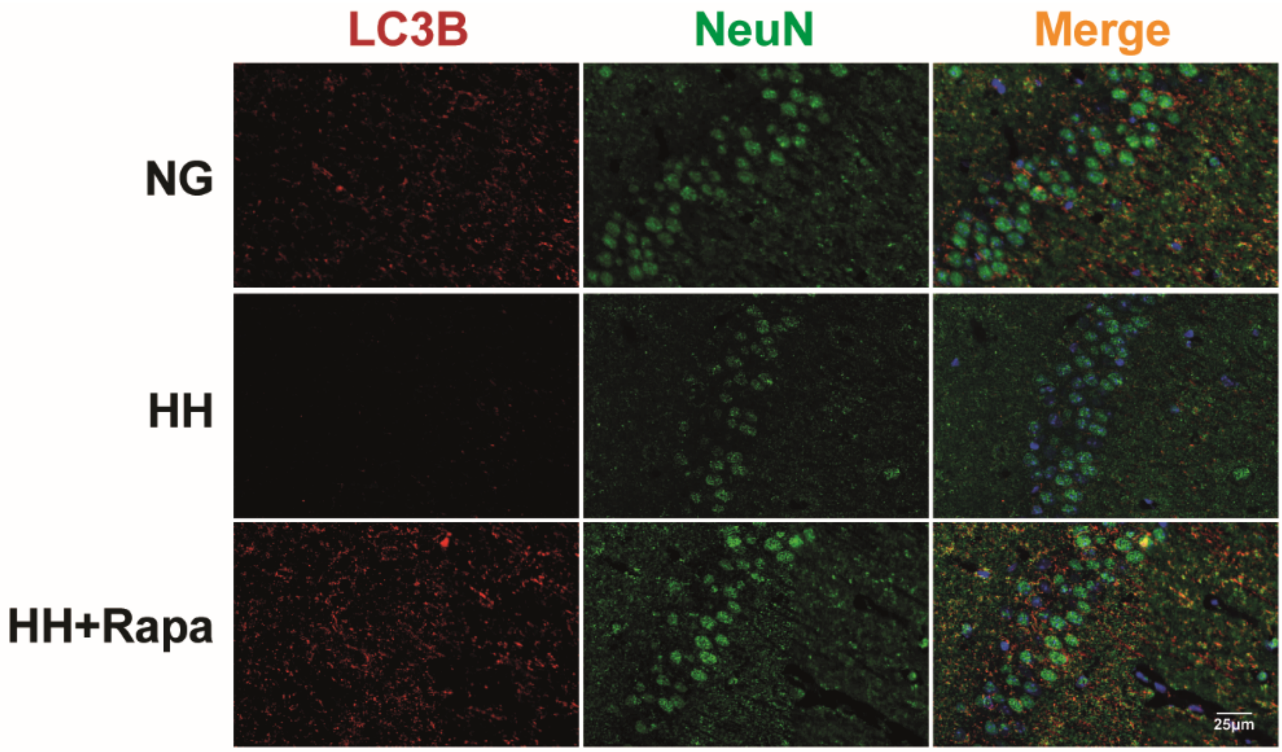

Fig. S5 Double-immunofluorescence staining indicates LC3B expression in hippocampal neurons after hypobaric hypoxia exposure of 6 km for 7 days. LC3B is marked as red puncta and neurons are marked green. The cells co-expressing LC3B and NeuN are merged as yellow puncta. Scale bar, 25 μm.

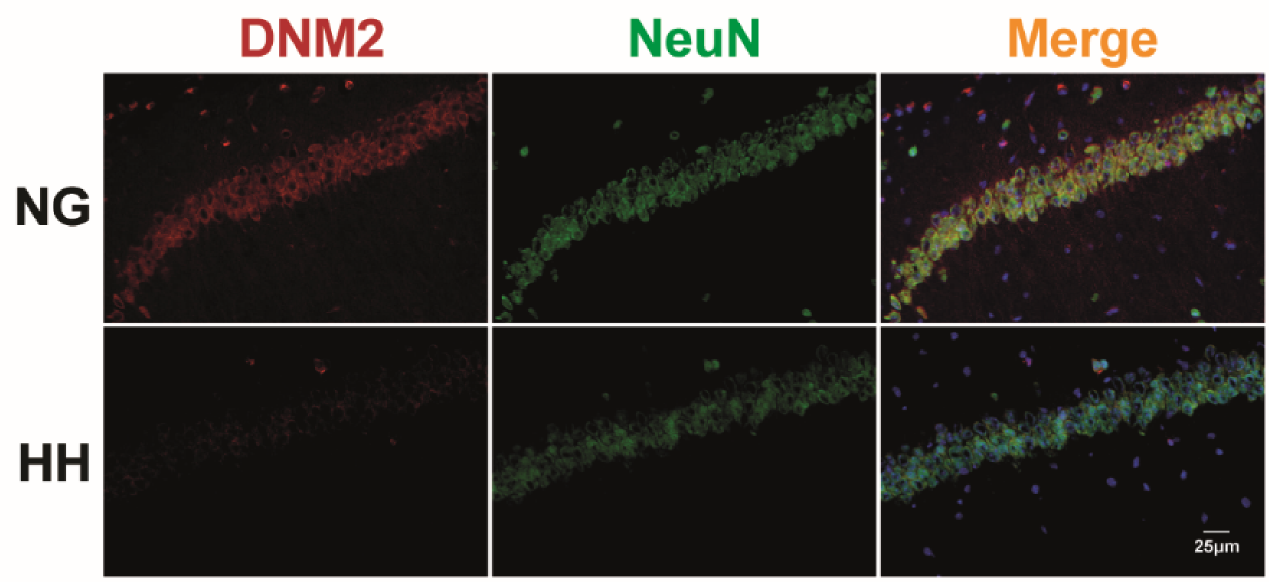

Fig. S6 Double-immunofluorescence staining indicates DNM2 expression (red) in hippocampal neurons (green) in mouse hippocampus after hypobaric hypoxia (6 km, 7 days). The cells co-expressing DNM2 and NeuN are merged as yellow puncta. Scale bar, 25 μm.

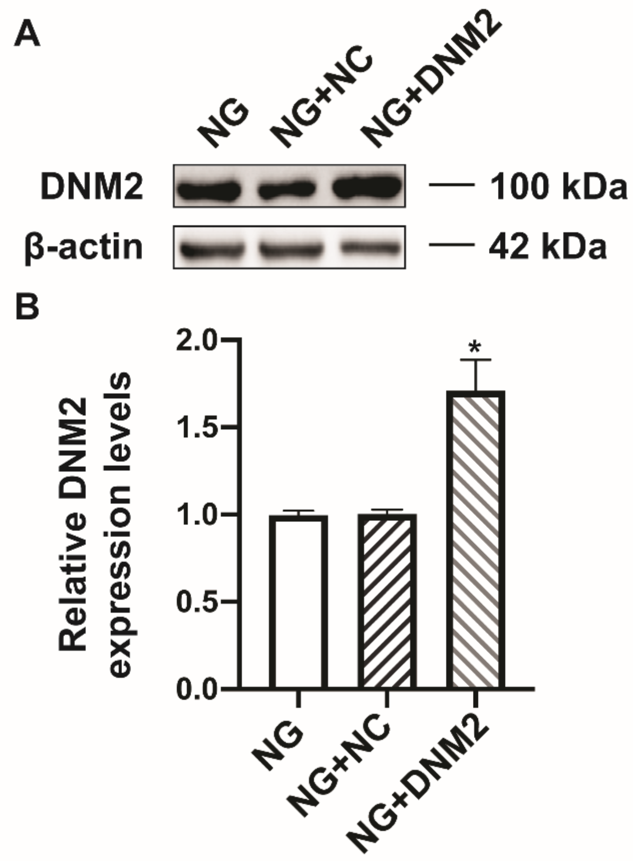

Fig. S7 The expression of DNM2 in the hippocampus of mice with AAV2/9 infection. **A** Western blots and analysis confirm the transfection effects of AAV2/9-hSyn-DNM2. **B** The relative integrated density of DNM2 to β-actin. Data are presented as the mean ± SD, $n = 6$, $^{*}P < 0.05$ *vs* NG+NC groups (one-way ANOVA followed by *post hoc* Tukey's test for multiple comparisons).

Table S1 Behavioral performance after HH exposure (data are shown as the mean ± SD)

**Groups**		Scores				
**Altitude (km)**	Time (day)	Escape latency (s)	Time in the target quadrant (s)	Visits to target quadrant (#)	Distance in the target quadrant (%)	Velocity (cm/s)
**NG (0)**	1	26.8 ± 5.8	34.9 ± 13.3	5.2 ± 1.3	53.8 ± 10.8	15.5 ± 4.6
	3	25.4 ± 6.2	37.7 ± 10.4	4.4 ± 1.3	57.0 ± 16.3	19.6 ± 5.4
	7	23.3 ± 4.3	44.5 ± 12.9	4.9 ± 1.5	62.1 ± 5.3	18.3 ± 3.7
**4**	1	30.0 ± 5.5	32.0 ± 6.5	4.4 ± 1.7	52.6 ± 5.2	15.6 ± 4.5
	3	33.7 ± 5.7	26.0 ± 4.8	3.3 ± 1.4	48.3 ± 7.1	10.6 ± 1.5
	7	48.3 ± 9.7	13.6 ± 9.0	1.8 ± 1.6	28.7 ± 17.1	9.0 ± 3.7
**6**	1	37.8 ± 9.0	30.1 ± 4.4	3.2 ± 1.6	47.0 ± 10.1	11.4 ± 3.2
	3	44.7 ± 8.2	17.7 ± 3.8	2.8 ± 1.3	40.6 ± 10.4	9.2 ± 1.7
	7	50.6 ± 11.3	8.8 ± 6.3	1.0 ± 1.4	22.1 ± 14.2	8.5 ± 1.3
**8**	1	44.0 ± 7.3	25.8 ± 9.3	2.6 ± 1.3	39.9 ± 8.1	8.6 ± 1.1
	3	51.8 ± 7.9	14.7 ± 11.7	1.3 ± 1.0	25.7 ± 16.6	7.2 ± 1.9
	7	59.1 ± 1.8	3.49 ± 3.8	0.2 ± 0.4	10.8 ± 11.7	5.8± 1.3

Table S2 Oxidative stress in the hippocampus after 6 km altitude exposure (data are shown as the mean ± SD)

Days	Oxidative stress-related proteins			
	ROS (fold change)	MDA (nmol/mg)	SOD (U/mg)	GSH-Px（mg/g）
**NG (0)**	1.0 ± 0.1	14.6 ± 2.1	7.6 ± 0.9	10.8 ± 1.0
**1**	2.6 ± 0.5	34.9 ± 5.2	3.9 ± 0.8	16.2 ± 1.9
**3**	3.4 ± 0.2	41.5 ± 2.1	2.5 ± 0.2	6.4 ± 0.7
**7**	4.0 ± 0.5	52.5 ± 2.1	2.5 ± 0.3	5.4 ± 0.8

Table S3 JC-1^{+} cell number in the hippocampus after 6 km altitude exposure (data are shown as the mean ± SD)

**number/mm^2**	Days			
	NG (0)	1	3	7
**FJC^{+} cell**	45.7 ± 16.8	60.8 ± 11.4	110.0 ± 17.5	187.2 ± 34.2

Table S4 Bcl-2/Bax protein expression in the hippocampus after 6 km altitude exposure (data are shown as the mean ± SD)

**Fold change**	Days			
	NG (0)	1	3	7
**Bcl-2/Bax**	3.0 ± 0.7	1.7 ± 0.5	0.8 ± 0.2	0.3 ± 0.2

Table S5 Protein expression in the hippocampus after 6 km altitude exposure (data are shown as the mean ± SD)

Group	Fold change			
	p-mTOR	P62	LC3B-II/I	LC3B-II
**NG**	1.0 ± 0.0	1.0 ± 0.1	1.0 ± 0.0	1.0 ± 0.0
**HH**	1.9 ± 0.1	1.6 ± 0.5	0.8 ± 0.1	0.5 ±0.1

Table S6 Autophagic structure in the hippocampus after 6 km altitude exposure (data are shown as the mean ± SD)

Group	Puncta/cell		
	AP	AL	AP+AL
**NG**	61.3 ± 10.5	47.2 ± 7.2	108.5 ± 9.5
**HH**	40.8 ± 10.3	29.2 ± 17.4	70.0 ± 23.7

Table S7 Protein expression in the hippocampus of mice treated with/without rapamycin after 6 km altitude exposure (data are shown as the mean ± SD)

Group	Fold change				
	p-mTOR	P62	LC3B-II/I	LC3B-II	DNM2
**NG**	1.0 ± 0.0	1.0 ± 0.1	1.0 ± 0.0	1.0 ± 0.0	1.0 ± 0.1
**HH+Veh**	1.9 ± 0.2	1.9 ± 0.4	0.5 ± 0.2	0.4 ± 0.1	0.3 ± 0.2
**HH+Rapa**	1.3 ± 0.1	3.0 ± 0.8	0.9 ± 0.1	0.8 ± 0.1	0.3 ± 0.1

Table S8 Autophagic structure in the hippocampus of mice after 6 km altitude exposure (data are shown as the mean ± SD)

**Group**	**Puncta/cell**		
	AP	AL	AP+AL
**HH+Veh**	35.8 ± 7.9	22.8 ± 15.7	58.7 ± 12.7
**HH+Rapa**	68.0 ± 18.6	25.0 ± 4.7	93.0 ± 19.1
**HH+Rapa+NC**	73.8 ± 19.2	25.0 ± 5.8	98.8 ± 17.9
**HH+Rapa+DNM2**	92.5 ± 14.6	51.7 ± 7.9	144.2 ± 17.6

Table S9 Protein expression in the hippocampus of mice with/without DNM2 overexpression after 6 km altitude exposure (data are shown as the mean ± SD)

**Group**	**Fold change**		
	P62	LC3B-II/I	LC3B-II
**NC**	1.0 ± 0.0	1.0 ± 0.0	1.0 ± 0.0
**NC+Rapa+Baf**	3.6 ± 0.5	3.1 ± 0.1	1.9 ± 0.2
**NC+Rapa**	2.3 ± 0.4	2.8 ± 0.2	1.8 ± 0.1
**DNM2**	0.6 ± 0.1	0.9 ± 0.1	1.1 ± 0.0
**DNM2+Rapa**	1.4 ± 0.3	1.5 ± 0.2	1.5 ± 0.1
**DNM2+Rapa+Baf**	3.9 ± 0.4	3.0 ± 0.1	1.7 ± 0.1

Table S10 Oxidative stress in the hippocampus of mice with/without DNM2 overexpression after 6 km altitude exposure (data are shown as the mean ± SD)

**Group**	**OS**			
	ROS (fold change)	MDA (nmol/mg)	SOD (U/mg)	GSH-Px (mg/g)
**NC+Veh**	1.0 ± 0.0	49.5 ± 4.5	2.4 ± 0.3	4.7 ± 0.5
**NC+Rapa**	0.6 ± 0.1	29.6 ± 5.5	4.1 ± 0.7	6.8 ± 1.2
**NC+3-MA**	0.9 ± 0.2	38.2 ± 3.7	2.1 ± 0.1	4.3 ± 0.7
**DNM2+Veh**	0.6 ± 0.1	41.7 ± 2.2	2.6 ± 0.3	5.4 ± 0.5
**DNM2+Rapa**	0.4 ± 0.1	19.8 ± 2.8	5.0 ± 0.7	8.4 ± 1.0
**DNM2+ 3-MA**	0.6 ± 0.1	30.0 ± 4.1	2.8 ± 0.4	6.1 ± 0.7

Table S11 JC-1^+ cell number in the hippocampus of mice with DNM2 overexpression after 6 km altitude exposure (data are shown as the mean ± SD)

**number/mm^2**	**Group**		
	Veh	Rapa	3-MA
**FJC$^+$ cell**	151.3 ± 22.5	85.0 ± 7.9	134.0 ± 7.0

Table S12 Bcl-2/Bax protein expression in the hippocampus of mice with DNM2 overexpression after 6 km altitude exposure (data are shown as the mean ± SD)

**Fold change**	**Group**		
	Veh	Rapa	3-MA
**Bcl-2/Bax**	0.4 ± 0.1	2.0 ± 0.2	1.1 ± 0.1

Table S13 Behavioral performance with/without DNM2 overexpression after 6 km altitude exposure (data are shown as the mean ± SD)

**Groups**	**Scores**				
	Escape latency (s)	Time in the target quadrant (s)	Visits to target quadrant (#)	Distance in the target quadrant (%)	Velocity (cm/s)
**NC+Veh**	48.2 ± 13.0	6.8 ± 6.0	0.8 ± 1.0	15.9 ± 8.4	8.8 ± 1.2
**NC+Rapa**	35.3 ± 13.0	15.7 ± 8.0	1.5 ± 1.0	24.0±7.8	14.1 ± 2.1
**NC+3-MA**	55.3 ± 8.8	7.1 ± 3.8	0.3 ± 0.5	21.0±4.2	8.6 ± 1.4
**DNM2+Veh**	45.2 ± 12.1	8.7 ± 4.8	0.8 ± 0.8	19.4 ± 10.3	8.6 ± 1.3
**DNM2+Rapa**	25.2 ± 5.2	32.2 ± 11.2	3.2±1.2	38.7 ± 4.9	15.6 ± 1.2
**DNM2+3-MA**	44.7 ± 8.5	15.5 ± 3.5	1.3±0.8	24.2 ± 4.1	8.5 ± 1.6
